# CO_2_ hydrogenation to methanol and dimethyl ether at atmospheric pressure using Cu-Ho-Ga/γ–Al_2_O_3_ and Cu-Ho-Ga/ZSM-5: Experimental study and thermodynamic analysis

**DOI:** 10.3906/kim-2009-66

**Published:** 2021-02-17

**Authors:** Cansu TUYGUN, Bahar İPEK

**Affiliations:** 1 Department of Chemical Engineering, Faculty of Engineering, Middle East Technical University, Ankara Turkey

**Keywords:** CO_2_hydrogenation to methanol, dimethyl ether, atmospheric pressure, holmium, gallium, thermodynamic analysis

## Abstract

CO_2_ valorization through chemical reactions attracts significant attention due to the mitigation of greenhouse gas effects. This article covers the catalytic hydrogenation of CO_2_ to methanol and dimethyl ether using Cu-Ho-Ga containing ZSM-5 and g-Al_2_O_3_ at atmospheric pressure and at temperatures of 210 °C and 260 °C using a CO_2_:H_2_ feed ratio of 1:3 and 1:9. In addition, the thermodynamic limitations of methanol and DME formation from CO_2_ was investigated at a temperature range of 100–400 °C. Cu-Ho-Ga/g-Al_2_O_3_ catalyst shows the highest formation rate of methanol (90.3 µmol_CH3OH_/g_cat_/h ) and DME (13.2 µmol_DME_/g_cat_/h) as well as the highest selectivity towards methanol and DME (39.9 %) at 210 °C using a CO_2_:H_2_ 1:9 feed ratio. In both the thermodynamic analysis and reaction results, the higher concentration of H_2_ in the feed and lower reaction temperature resulted in higher DME selectivity and lower CO production rates.

## 1. Introduction

CO_2_ emissions to the atmosphere increased 15 billion metric tons from 1990 till 2020 because of the increasing fossil fuel usage in industry, transportation, and electricity generation. After the 2000s, a decreasing trend in CO_2_ emissions was observed due to the uses of more environmentally friendly industrial technologies1Sources of Greenhouse Gas Emission [online]. Website https://www.epa.gov/ghgemissions/sources-greenhouse-gas-emissions?sa=X&ved=2ahUKEwjYjN_N8KDlAhVLUxoKHaQSBXsQ9QF6BAgLEAI. [accessed 01 July 2020]; however, CO_2_ concentration in the atmosphere continues to increase (to a value of 410 ppm (v/v) in 20202Earth’s Home Page [online]. Website https://www.CO_2_.earth/. [accessed 01 July 2020]). As reported by Intergovernmental Panel on Climate Change (IPCC), the concentration of CO_2_ in atmosphere is predicted to be nearly 600 pmmv in 20503Carbon Dioxide: Projected emissions and concentrations [online]. http://www.ipcc-data.org/observ/ddc_CO_2_.html. [accessed 01 July 2020] .

There are several methods for CO_2_ removal from the atmosphere such as photosynthesis or biological sequestration by plants and microorganisms and carbon capture and sequestration (CCS) technology4Cho R. (2018) Removing Carbon from the Atmosphere Save Us from Climate Catastrophe [online]. Website https://blogs.ei.columbia.edu/2018/11/27/carbon-dioxide-removal-climate-change/ [accessed 01 July 2020] . CCS technology can reduce up to 90% of the carbon dioxide emissions from power plants; however, CCS is considered to be only a temporary solution. Instead, efficient utilization of CO_2_ in chemical industry is considered to be a more desired solution. Hydrogenation of CO_2_ to methanol is particularly important considering the potential of methanol as a feedstock in production of various chemicals such as formaldehyde, dimethyl ether (DME), ethanol, olefins, and formic acid [1]. CO_2_ hydrogenation not only reduces the cost of CO_2_ disposal, but also provides easier transportation and easier storage through liquid methanol production [2]. There are two main reactions considered for methanol production through CO_2_ hydrogenation (see Eq. (1) and (2)).

(1)CO2+2H2⇋CH3OH+H2OΔH0(298K)=-49.5kJ/mol

(2)CO2+H2⇋CO+H2OΔHO(298K)=41.2kJ/mol

CO_2_ can be hydrogenated to produce carbon monoxide or methanol under relatively high-pressure conditions and mild temperatures using Cu-based catalysts. Cu-based traditional catalyst, i.e. Cu/ZnO/Al_2_O_3_ is used in methanol production from CO_2_ hydrogenation at a temperature range of 200–300 °C and pressures between 50 bar and 100 bar [3–6]. However, it is desirable to efficiently conduct the carbon dioxide hydrogenation reaction at lower pressures to decrease the cost of the process. Under low-pressure conditions, selection of a catalyst is crucial to achieve desirable kinetics. Since Cu/ZnO/Al_2_O_3_ catalyst is not active under low-pressure conditions [7], the rate of methanol formation decreases significantly under atmospheric conditions. For instance, the methanol formation rate was obtained as 1.74 μmol_MeOH_/g_cat_/min at 190 **°** C and 1 atm by using Cu/ZnO catalyst [8]. At atmospheric pressure and high temperatures (higher than 300 °C), Ru, Ni, Fe, and Co catalysts can produce higher production rates of methanol [9]. Ni_5_Ga_3_/SiO_2_ catalyst, which is prepared by impregnation method, is more advanced when compared with Cu-based catalyst, reaching a methanol production activity of 125 μmol_MeOH_/g_cat_/min at 210 **°** C and 1 atm (CO_2_:H_2_ = 1:3) [10].

The low methanol selectivity is one of the main problems at the atmospheric pressure due to the dominant reverse water gas shift reaction. Hence, activity of rWGS reaction should be suppressed to achieve high methanol selectivity values. Cu-based (Cu/ZnO) catalyst, which gives lower methanol selectivity, has higher activity for rWGS reaction compared to the Ni-Ga–based catalyst under low-pressure conditions [9,11,12]. According to Díez-Ramírez, 35% methanol selectivity (and a methanol formation rate of approximately 1.2 μmol_MeOH_/g_cat_/min) was obtained from the activity of Cu/ZnO catalyst at 210 **°** C and atmospheric pressure with a CO_2_:H_2_ ratio of 1:9 [11]. The catalyst Ni_5_Ga_3_ was reported to show 42.2 μmol_MeOH_/g_cat_/min methanol formation rate at 200 **°** C and atmospheric pressure using a CO_2_:H_2_ ratio of 1:9 [13]. At a higher temperature, i.e. at 230 **°** C, and at atmospheric pressure, a methanol and dimethyl ether selectivity of approximately 2.5% was observed on Cu/ZnO/Al_2_O_3_ whereas methanol and dimethyl ether selectivity increased to approximately 15% on Ni_5_Ga_3_/SiO_2_ under the same conditions [9]. Although Ni-rich site performs in both rWGS and methanol production reactions, there is the limitation for poisoning Ni site by adsorbed CO and carbon atoms. Gallium-rich site is reported to be responsible for methanol and DME synthesis reaction due to its effect on providing higher selectivity (%) of both methanol and DME at a temperature range of 200–240 **°** C and at 1 atm using a CO_2_:H_2_ ratio of 1:3 [9]. According to Zohour et al. [14] a potential trimetallic catalyst combination for methanol production is Cu-GaOx-HoOy impregnated on g–Al_2_O_3_. A combined rate for methanol and dimethyl ether production of 1.6 × 10–4 s–1 is reported on Cu-GaOx-HoOy/g–Al_2_O_3_ at 1 atm and 260 **°** C (CO_2_:H_2_ ratio is 1:3) compared to a turnover frequency of 0.45 × 10–5 s–1 observed on Cu-ZnO/g–Al_2_O_3_ [14]. 

The low methanol and high CO formation rates reported in the literature for atmospheric pressure conditions can be explained by the thermodynamic analysis of the reactions (1) and (2) at atmospheric pressure [13]. According to the reported thermodynamics analysis, the methanol selectivity decreased from 48% to 0.1% from 127 °C to 277 °C, while CO_2_ conversion could only reach 20% at 277 °C (from 8% at 127 °C ) [13]. On the other hand, at elevated pressures (P = 50 bar), the equilibrium conversion of CO_2_ could reach 45% at 212 °C (with methanol selectivity of 17.5%) [15]. 

The thermodynamic limit on the CO_2_ conversion and methanol selectivity is often tackled by combining the methanol formation with methanol dehydration (Eq. (3)) to form dimethyl ether [15,16]. According to our knowledge, there is no study in the literature on the thermodynamic analysis of CO_2_ hydrogenation to methanol and dimethyl ether considering the reactions 1, 2, and 3 at atmospheric pressure.

(3)2CH3OH⇋CH3OCH3+H2OΔHO(298K)=-23.6kJ/mol

When methanol production catalysts are combined with solid acid catalysts such as g–Al_2_O_3_ or zeolites, the catalysts become bifunctional for both methanol production and dimethyl ether synthesis. Firstly, methanol is produced on metal-based catalysts, which is then dehydrated to dimethyl ether on solid acid catalysts. The types of solid acid catalyst that can produce dimethyl ether at atmospheric pressure and nearly at 260–300 **°** C are commercial g–Al_2_O_3_ or H^+^-zeolites such as H^+^–ZSM-5 zeolite [17–19], which is more acidic than g–Al_2_O_3_. Since the water produced on the methanol dehydration reaction has an inhibition effect on acid sites of g–Al_2_O_3_ [20], zeolites are often preferred due to the stability of the zeolites in the presence of water [21]. The active centers for dimethyl ether synthesis in zeolites are Bronsted acid centers [22]. Depending on the pore structure of zeolite, the formation of different hydrocarbons and coke apart from methanol and dimethyl ether can be observed. This can be caused by Bronsted acid centers having different activities. 

In this work, the thermodynamic limitations of methanol and dimethyl ether formation from carbon dioxide are studied at the atmospheric pressure. The thermodynamic analysis for CO_2_ hydrogenation to methanol and dimethyl ether is performed using a temperature range of 100–400 °C and CO_2_:H_2_ feed ratios of 1:3 and 1:9. We also examined the effect of H+-ZSM-5 and g-Al_2_O_3_ as solid acid catalysts including a ternary metal catalyst: Cu-Ho-Ga for carbon dioxide hydrogenation reaction at the atmospheric pressure and temperatures between 210 °C and 260 °C.

## 2. Materials and methods

### 2.1. Thermodynamic considerations on CO_2_ hydrogenation to DME process

The thermodynamic analysis of CO_2_ hydrogenation to methanol and DME at atmospheric pressure was performed using numerical methods in this work. The equilibrium constants are calculated for CO_2_ hydrogenation, reverse water gas shift reaction and methanol dehydration reactions (see Eqs. (1)–(3)) using Tables 1 and 2 and Eqs. (4)–(7). *K* *j* represents the equilibrium constant of reaction *j* , *P* *i* represents the partial pressure of species *i* , *ν* *ij* represents the stoichiometric coefficient of species *i* in reaction *j* . Δ *Ğ* *rj* represents the Gibbs free energy of reaction *j* calculated at the specific temperature. The molar heat capacity at constant pressure of the species *i* (cp, *i* , J/mol/K) is shown in Eq. (8), for which the constants A, B, C, and D can be found in Table 2. 

(4)Kj(T)=∏iPivij=exp(-ΔGˇrjRT)

(5)dlnKjdT=Δrxn.jHTRxT2=Δrxn.jH298K+∫T=TrefT=TΔcp.jdTRxT2

(6)Kj=Kj,298xexp[-constantR(1T-1Tref)+ΔArxn,jRxln(TTref)+Δ Brxn.j2xR*(T-Tref)+δ Crxn.j6cR*(T2-Tref2)Δ Drxn.j12xR*(T3-Tref3)]

(7)constant=Δrxn.jH298K-ΔArxn.jxTref-ΔBrxn.jTref22-ΔCrxn,jTref33-ΔDrxn,jTref44

(8)cp,i=Ai+BixT+CiT2+DixT3 and δcp,j=∑cp.ivij

**Table 1:** Considered reactions and the thermodynamic constants.

Rxn no.	Reaction stoichiometry	∆H°298K (kJ/mol)	∆S°298K (kJ/mol.K)	∆G°298K (kJ/mol)	Extent of rxn (xi)	Keq
1		–49.5	–0.178	3.574	x1	K1
2		41.20	0.042	28.684	x2	K2
3		–23.6	–0.022	–17.004	x3	K3

**Table 2:** Thermodynamic parameters used for equilibrium constant calculations [37,38].

Parameters	CO_2_(i = 1)	H_2_(i = 2)	CO(i = 3)	CH3OH (i = 4)	H_2_O (i = 5)	CH3OCH3(i = 6)
A (J/mol)	27.4730	25.3990	25.5560	40.0460	33.9330	22.6370
B (×10–3)	42.3150	20.1780	–6.5807	–3.8287	–8.4186	164.8000
C ((×10–5)	–1.9555	–3.8549	2.0130	24.5290	2.9906	–5.0000
D ((×10–8)	3.9968	31.8800	–12.2270	–216.7900	–17.8250	0.3000
∆fH_2_98K (kJ/mol)	–393.500	0	–110.500	–201.170	–241.800	–184.100
∆fS298K (kJ/mol/K)	0.214	0.131	0.198	0.240	0.189	0.269

The mole fractions and selectivity values are calculated based on the extend of the reactions (see Table 3, Eq (21)).

(9)K1=yCH3OHxyH2=p2xyCO2xyH23

(10)K2=yCOxyH2OyCO2xyH2

(11)K3=yCH3OCH3xyH2OyCH3O2

(12)yCO2=nCO2-ξ1-ξ2nCO2+nH2-2xξ1

(13)yH2=nH2-3xξ1-ξ2nCO2+nH2-2xξ1

(14)yCO=ξ2nCO2+nH2-2xξ1

(15)yH2O=ξ1+ξ2+ξ3nCO2+nH2-2xξ1

(16)yCH3OH=ξ1-2xξ3nCO2+nH2-2xξ1

(17)yCH3OCH3=ξ3nCO2+nH2-2xξ1

(18)SDME=2xξ3ξ1+ξ2

(19)SCH3OH=ξ1-2xξ3ξ1+ξ2

(20)SCO=ξ2ξ1+ξ2

(21)XCO2=ξ1+ξ2nCO2

**Table 3:** Extent of reactions.

Reaction no.	CO_2_	H_2_	CO	CH3OH	H_2_O	CH3OCH3
1	-x1	-3x1	-	+x1	+x1	-
2	-x2	-x2	+x2	+x2	-	-
3	-	-	-	+x3	-2x3	+x3
total	nCO_2_-x1-x2	nH_2_-3x1-x2	x2	x1+x2+x3	x1-2x3	x3

### 2.2. Experimental procedures

#### 2.2.1. Catalyst preparation

##### 2.2.1.1. Materials for zeolite synthesis

In zeolite synthesis, colloidal silica LUDOX HS–40 (Aldrich, 40 wt.%, liquid) was used as the silicon source, sodium aluminate (NaAlO_3_) (Sigma-Aldrich, 55 wt.% Al_2_O_3_, 44 wt.%, solid) was used as the aluminum source and tetrapropylammonium hydroxide (TPAOH) (Merck, 40 wt.%, liquid) was used as the structure-directing agent (SDA). For NH_4_^+^-exchange of ZSM-5, NH_4_NO_3_ (Sigma-Aldrich, 99 wt.%, solid) was used as the NH_4_^+^ source. 

##### 2.2.1.2. Synthesis of ZSM–5

ZSM–5 zeolite with Si/Al molar ratio is equal to 72 was synthesized hydrothermally using a procedure given by Mei et al. [23]. In the synthesis procedure, 9.654 g of TPAOH was used as the structure directing agent. The molar composition of the synthesis gel mixture was 140SiO_2_:1Al_2_O_3_:8TPAOH:800H_2_O. During the synthesis, two solutions were prepared separately. For the first solution, 0.441 g of sodium aluminate and 4.830 g of TPAOH were added in 17.663 g of water. This first solution was stirred for 45 min at room temperature using 300 rpm stirring rate. In another flask, 49.920 g of Ludox LS–40 and 4.830 g of TPAOH were stirred to prepare the second solution at room temperature using 300 rpm stirring rate. After 45 min, the first solution was added to the second solution dropwise. The mixed solution was stirred for 7 h at room temperature using 300 rpm stirring rate. Two autoclaves with Teflon liners (35 mL) were filled with this solution and the hydrothermal synthesis was conducted at 180 °C for 5 days under static conditions. The synthesized zeolite crystals were vacuum filtered using cellulose acetate membrane filter that has 0.2 µm pore diameter and then washed with deionized (DI) water. The mass of ZSM–5 was 19 g after the drying. All samples were dehydrated at 120 °C for 1 h and calcined at 550 °C for 5 h using a 1 °C per min heating rate in a muffle furnace. Then, 5 g of zeolite was exchanged in 0.2 M NH_4_NO_3_ solution (500 mL DI water) at 80 °C for 3 h using 300 rpm stirring rate. This exchange procedure was repeated 3-times to provide full NH_4_^+^ exchange. After the exchange procedure, NH_4_^+^-ZSM-5 was heated at 550 °C for 5 h using a 2 °C per min heating rate following drying at 120 °C for 1 h to obtain H+-ZSM-5.

##### 2.2.1.3. Materials for metal loading

For metal loading using incipient wetness method, 1.143 g of Cu(II)nitrate trihydrate (Merck, solid), 0.412 g of Ho(III)nitrate pentahydrate (Sigma Aldrich, 100 wt.%, solid), and 1.551 g of Ga(III)nitrate hydrate (Sigma Aldrich, 99.9 wt.%, solid) were used.

##### 2.2.1.4. Catalyst preparation by Cu-Ho-Ga loading for CO_2_ hydrogenation reaction

After the heat treatment, the amount of DI water that can be absorbed by 1 g of H^+^-ZSM-5 (Si / Al=72) was determined (0.8 mL). The metal concentrations to be loaded on support materials were determined to be: 10 wt.% Cu, 5 wt.% Ho, and 5 wt.% Ga. For 3 g of zeolite, 1.143 g of Cu(II) nitrate trihydrate, 1.551 g of Ga(III) nitrate hydrate, and 0.412 g of Ho(III) nitrate pentahydrate were dissolved in 2.4 mL of DI water consecutively. Prepared metal nitrate solution was absorbed by 3 g of H^+^–ZSM-5 (Si/Al = 72). After the H^+^-ZSM-5 was dried at 120 °C for 3 h, calcination was performed at 400 °C for 4 h using a 2 °C per min heating rate following drying at 120 °C for 1 h. This metal loading procedure was repeated for 3 g g–Al_2_O_3_ in 5.1 mL of DI water. The final samples were pelleted and sieved to result in particle sizes between 200 and 450 µm. 

#### 2.2.2. Characterization tests

The characterization of the prepared samples was performed using X-ray powder diffraction, N_2_ adsorption at –196 °C, and scanning electron microscopy. X-ray diffraction (XRD) was performed using a diffractometer (Rigaku Ultima IV, located at the Central Laboratory, Middle East Technical University) using a K-a Cu source (l = 1.5418 Å) operated at 40 kV and 30 mA. The diffractogram was obtained between 2q angles of 5-50° and the scanning speed was 1° per min. The images of the prepared ZSM-5 were collected using scanning electron microscopy (QUANTA 400F Field Emission SEM located at the Central Laboratory, Middle East Technical University). The elemental analysis is based on the energy dispersive X-ray spectroscopy analysis (EDX) taken from at least 5 different spots at an accelerating voltage of 30 kV. The textural properties of synthesized and metal-loaded ZSM-5 and metal-loaded g-Al_2_O_3_ were investigated using N2 adsorption at –196 °C. Prior to N_2_ (Oksan, 99.999%) adsorption experiments, the samples were degassed at 300 °C for 6 h. The adsorption experiments were performed at relative pressure values of 10^-5^ to 0.98 following dead volume measurements using He (Oksan, 99.999%). The temperature of the samples was maintained at –196 °C using liquid N_2_ in a dewar. The micropore volumes of the samples were calculated using statistical thickness, t-plot method.

H_2_ TPR experiments were performed on air-exposed calcined samples without any in situ dehydration to be consistent with the reduction procedure prior to the kinetic analysis. Three hundred and fifty milligrams of sample was loaded into a quartz reactor with an inner diameter of 7 mm and an outer diameter of 9 mm. The temperature of the reactor was increased to 625 °C from 30 °C using a heating rate of 5 °C per min. The effluent H_2_ was analyzed using an online GC (Agilent 7820A) equipped with a thermal conductivity detector (TCD) and a PoraPlot Q column (CP7554, 25 m × 0.530 mm × 20 mm) every 4 min.

XPS analysis was performed using K-Alpha XPS spectrometer (ThermoFisher Scientific, located at the National Nanotechnology Research Center, Bilkent University) following dehydration of calcined and spent samples at 300 °C for 5 h.

#### 2.2.3. Apparatus used for CO_2_ hydrogenation in catalytic reaction

**Figure 1:**
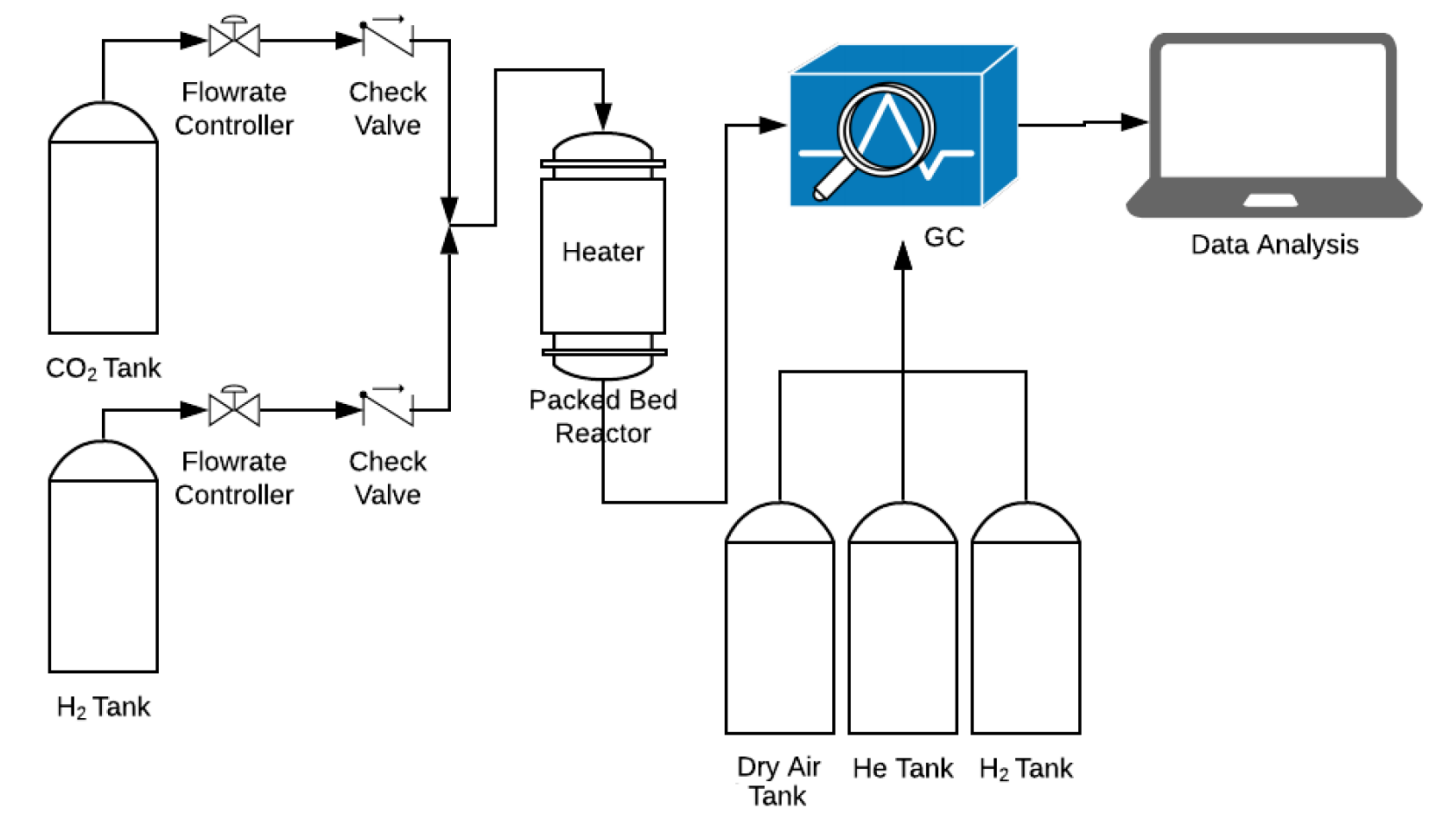
Experimental reaction setup.

The reaction setup can be seen in Figure 1. The gas flows were controlled using digital mass flow controllers (Alicat, MC-100SCCM-D/5M). A glass bubble flowmeter was used to measure the gas volumetric flow rates at the exit of the reactor. A quartz tube with an inner diameter of 7 mm and an outer diameter of 9 mm was used as the reactor. In this reactor, 1.7 cm bed length and 350 mg zeolite was loaded and supported by glass wool. A cylindrical furnace around the reactor tube was used as a heater and was controlled by a thermocouple attached to the center of the zeolite outside the quartz cylinder tube in a spiral fashion. The quantitative analysis of effluent gas of the reactor was performed using a gas chromatograph (GC) (Agilent 7820A). The carrier inert gas of GC was He together with H_2_ and dry air for flame ionization detector (FID). A PoraPlot Q column (CP7554, 25 m × 0.530 mm × 20 mm) was used for product separation. A flame ionization detector (FID) was used for element qualification and quantification. The split ratio of GC set to 1/5. The temperature of the GC oven was set initially at 40 °C with 5 min holding time in order to separate the light gases. Then the temperature of the oven inside the GC was set to rise from 40 °C to 160 °C at a 10 °C per min ramping rate with 3 min holding time, giving a total of 20 min method time. The sample was taken every 20 min using an autosampling valve with an inlet temperature of 150 °C and FID temperature of 300 °C.

#### 2.2.4. Reaction procedure for CO_2_ hydrogenation

The reaction procedure included two steps. The first step was reduction of catalysts using 50 sccm He and 50 sccm H_2_. The temperature of the reactor was set to 250 °C using a 5 °C per min heating rate. The temperature was then held at 250 °C for 2 h for reduction. In the second step of CO_2_ hydrogenation, CO_2_ flow was set to 25 sccm and H_2_ flow was set to 75 sccm in order to provide a CO_2_:H_2_ ratio of 1:3 or 10 sccm vs 90 sccm for a CO_2_:H_2_ ratio of 1:9 (The total volumetric flow rates were measured as 115 cm3 per min at 25 °C). The reaction was performed at 210 °C, the temperature was set to 210 °C using a heating rate of 5 °C per min. The reaction was performed for 2 h while taking online samples every 20 min. The reaction was also repeated at 260 °C with a CO_2_:H_2_ ratio of 1:3 and 1:9. The selectivity values and CO_2_ conversion were calculated based on equations given in Eqs. (22)–(25), where F_CO_2__ is the molar flow rate of CO_2_ in µmol/h.

(22)Selectivity,SCH3OH,%=rCH3OHrCH3OH+2xrDME+rCH4rCO

(23)Selectivity,SDME%=2xrDMErCH3OH+2xrDME+rCH4+rCO

(24)Selectivity,SCO%=rCOrCH3OH+2xrDME+rCH4+rCO

(25)Conversion, XCO2,%=rCH3OH+2xrDME+rCH4+rCOFCO2/gcat

## 3. Results and discussion

### 3.1. Thermodynamic analysis

**Figure 2:**
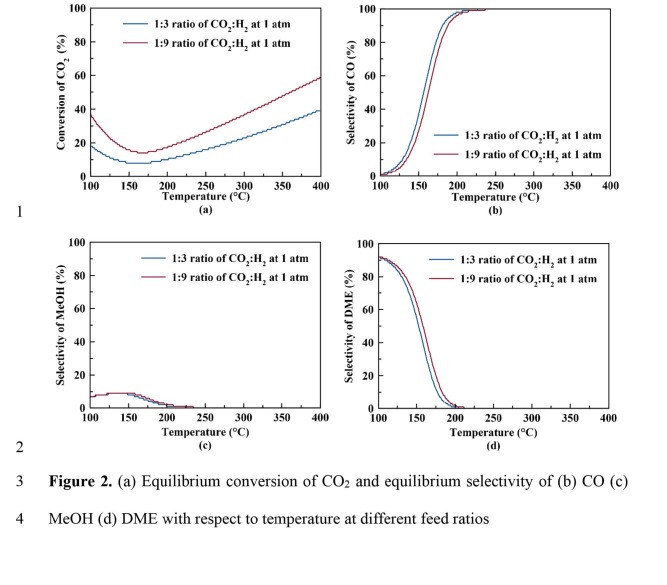
(a) Equilibrium conversion of CO_2_ and equilibrium selectivities of (b) CO, (c) MeOH, and (d) DME with respect to temperature at different feed ratios.

**Figure 3:**
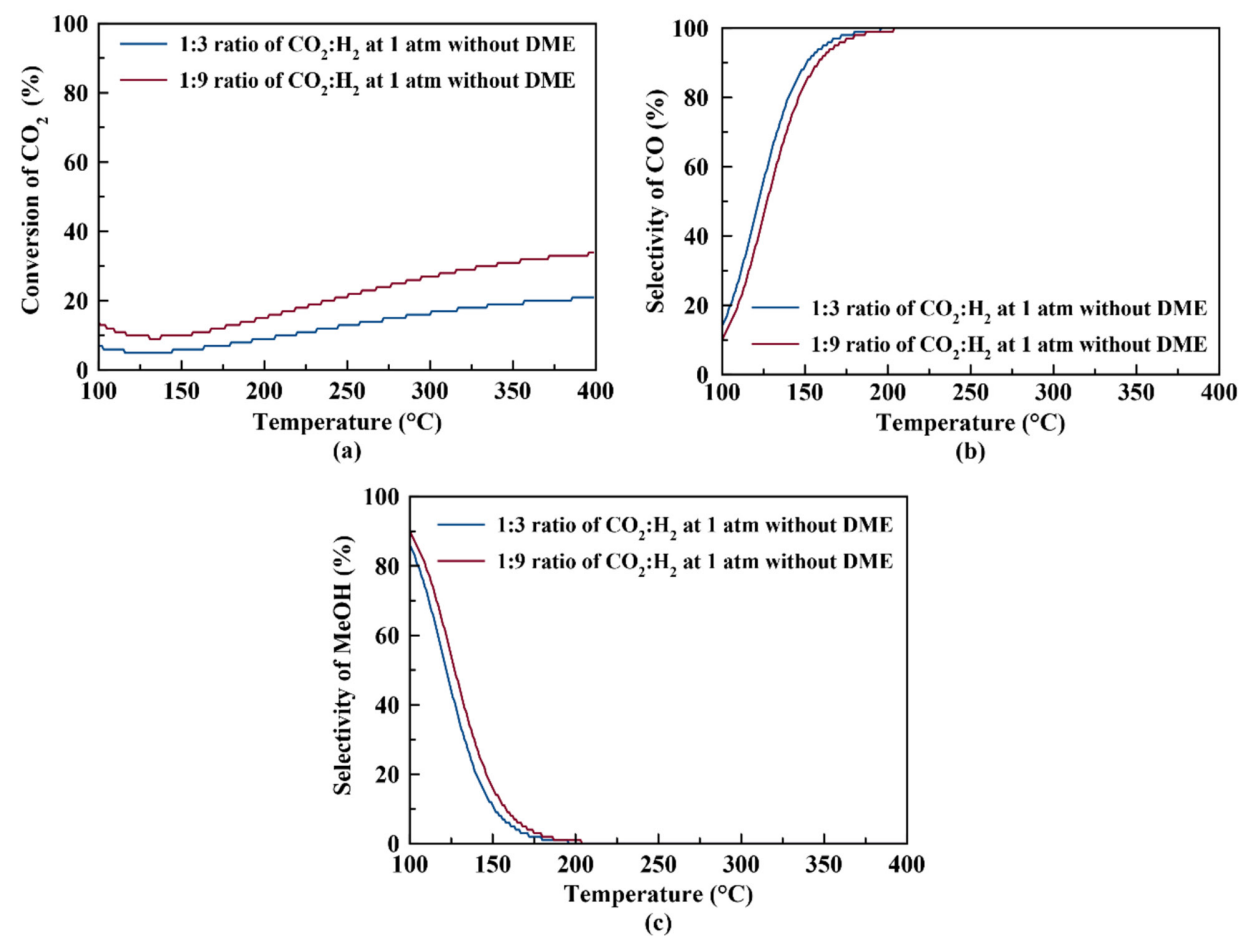
(a) Equilibrium conversion of CO_2_ and equilibrium selectivities of (b) CO and (c) MeOH with respect to temperature at different feed ratios without considering DME reaction.

The thermodynamic analyses were performed for CO_2_ hydrogenation with or without dimethyl ether production (see Figures 2a–2d and 3a–3c). Figure 2a represents the equilibrium conversion of CO_2_ to methanol and dimethyl ether with respect to temperature using different feed ratios. According to Figure 2a, higher CO_2_ conversion can be achieved at atmospheric pressure when CO_2_:H_2_ ratio is 1:9 (when compared to 1:3 CO_2_:H_2_ ratio). The equilibrium CO_2_ conversion increases from 17.3% to 28.3% at 260 °C for a CO_2_:H_2_ ratio of 1:9 when DME production is considered. On the other hand, when DME production is not taken into account (see Figure 3a), the equilibrium CO_2_ conversion increases from 13.6% to 22.6% at 260 °C when CO_2_:H_2_ ratio increases from 1:3 to 1:9.

Figure 2b represents the equilibrium selectivity of CO with respect to temperature using different feed ratios. According to Figure 2b, when CO_2_:H_2_ ratio is 1:9, selectivity of CO is less than that for CO_2_:H_2_ ratio of 1:3, indicating less favorable reverse water gas shift reaction at higher H_2_ ratios. A similar case is observed in the absence of dimethyl ether (see Figure 3b). Figure 2c represents the equilibrium selectivity of methanol (MeOH) with respect to temperature with different feed ratios. Slightly higher methanol selectivity could be achieved when CO_2_:H_2_ ratio is 1:9; nevertheless, the equilibrium methanol selectivity is very low (<1%) at temperatures higher than 200 °C. Figure 2d represents the equilibrium selectivity of DME with respect to temperature with different feed ratios, again showing a higher selectivity value when CO_2_:H_2_ ratio is 1:9. Both methanol and dimethyl ether selectivity values practically reach zero as temperature is increased to values higher than 250 °C.

According to the thermodynamic analysis, higher CO_2_ conversion, higher methanol selectivity and higher dimethyl ether selectivity can be achieved when H_2_ is used in higher concentrations in the feed. This is expected due to the higher H_2_ stoichiometric coefficient in methanol formation when compared to reverse water gas shift reaction.

**Figure 4:**
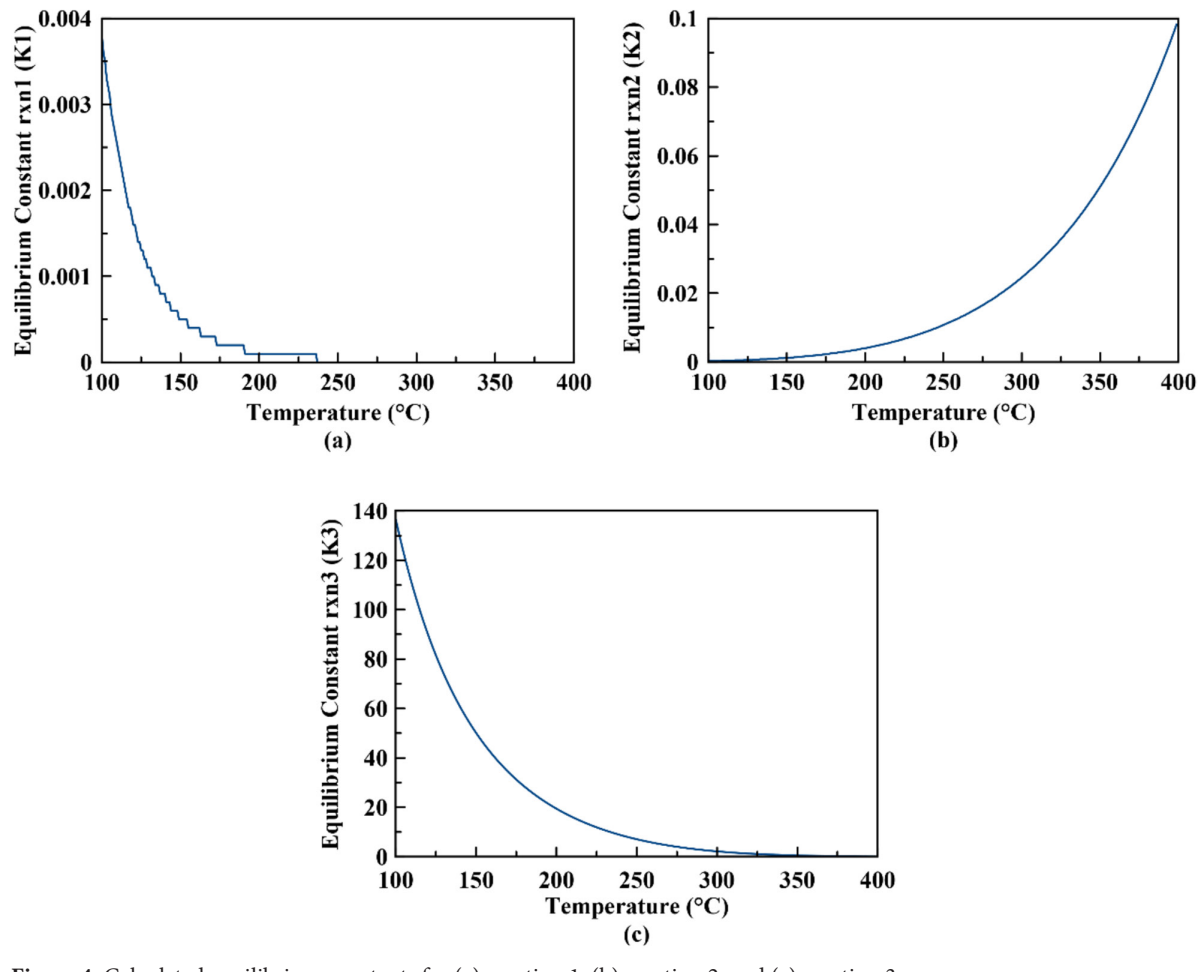
Calculated equilibrium constants for (a) reaction 1, (b) reaction 2, and (c) reaction 3.

At low temperatures, DME and methanol selectivity are higher than those at high temperatures. This is due to the fact that the equilibrium constants for reactions 1 and 3 are decreasing at higher temperatures (due to exothermic nature of the reactions) when compared to increasing equilibrium constant for CO production (Figure 4). This results in increasing CO production and decreasing methanol and DME production at higher temperatures.

When equilibrium results containing methanol dehydration (dimethyl ether production) are compared to those found by Ahmad et al. for CO_2_ hydrogenation to methanol without dimethyl ether production [13], a similar trend of higher methanol selectivity is observed at low temperatures at atmospheric pressure. When production of DME reaction is also considered at atmospheric pressure, methanol selectivity shows a maximum selectivity of 9.3% at 142 °C and then decreases with higher temperatures (see Figure 2c). When the DME reaction is not taken into account, methanol selectivity shows a steadily decreasing trend (Figure 3c). This may be due to the higher values of equilibrium constant of the DME production reaction when compared to those of the other reactions, as shown in Figure 4. Due to the lowest minus Gibbs free energy in the third reaction, the equilibrium constant of 3rd reaction gets higher values and therefore increasing the DME selectivity.

### 3.2. Characterization of catalysts

#### 3.2.1. XRD powder patterns

**Figure 5:**
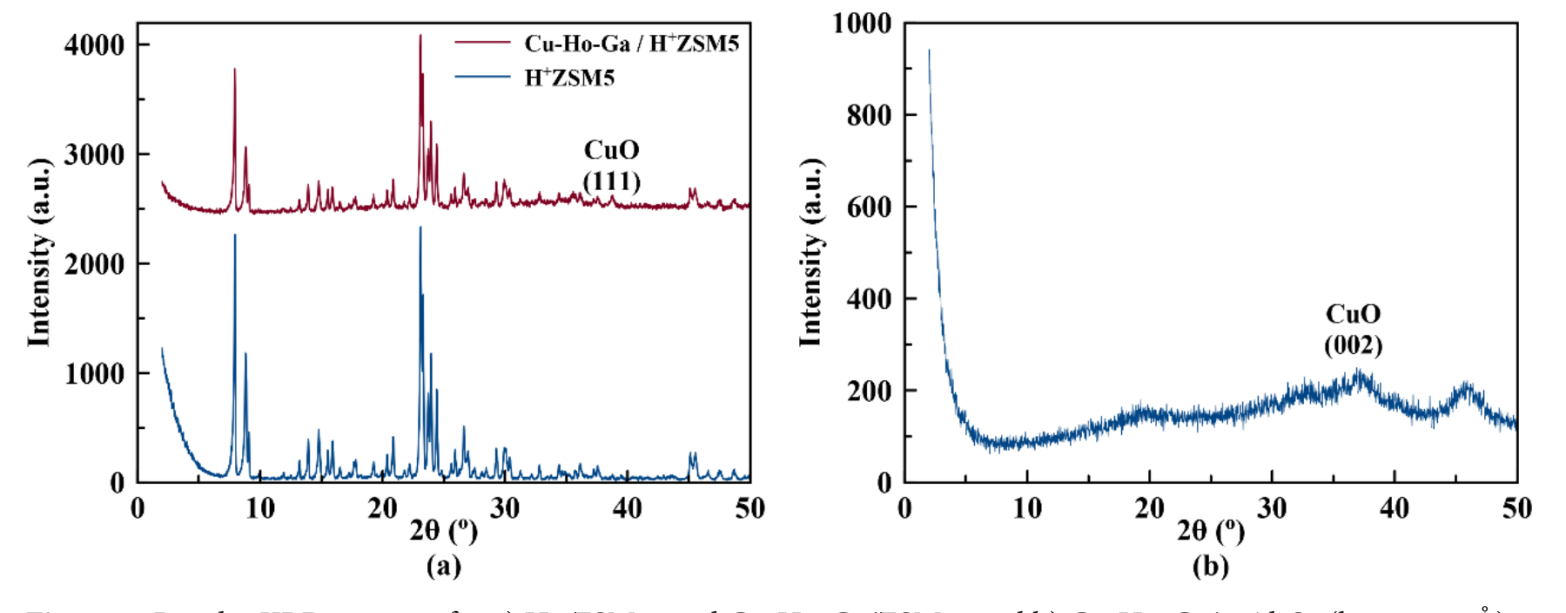
Powder XRD patterns for a) H^+^-ZSM-5 and Cu-Ho-Ga/ZSM-5 and b) Cu-Ho-Ga/g-Al_2_O_3_ (l = 1.5418 Å).

Powder X-Ray diffraction patterns for H+-ZSM-5 and Cu-Ho-Ga/ZSM-5 following calcination are shown in Figure 5a and the pattern for Cu-Ho-Ga/g-Al_2_O_3_ is shown in Figure 5b. H+-ZSM-5 was observed to have high crystallinity with signature peaks for MFI framework as expected. In XRD patterns of Cu-Ho-Ga/ZSM-5 and Cu-Ho-Ga/g-Al_2_O_3_, formation of CuO can be observed following Cu-Ho-Ga loading and calcination in air. Presence of CuO was determined on Cu-Ho-Ga/ZSM-5 and Cu-Ho-Ga/g-Al_2_O_3_ from the diffraction peak observed at the 2q angle of 38.7° (belonging to CuO (111) [24]) and at the 2q angle of 35.6° (belonging to CuO (002)[25]), respectively. 

#### 3.2.2. N2 adsorption tests

**Figure 6:**
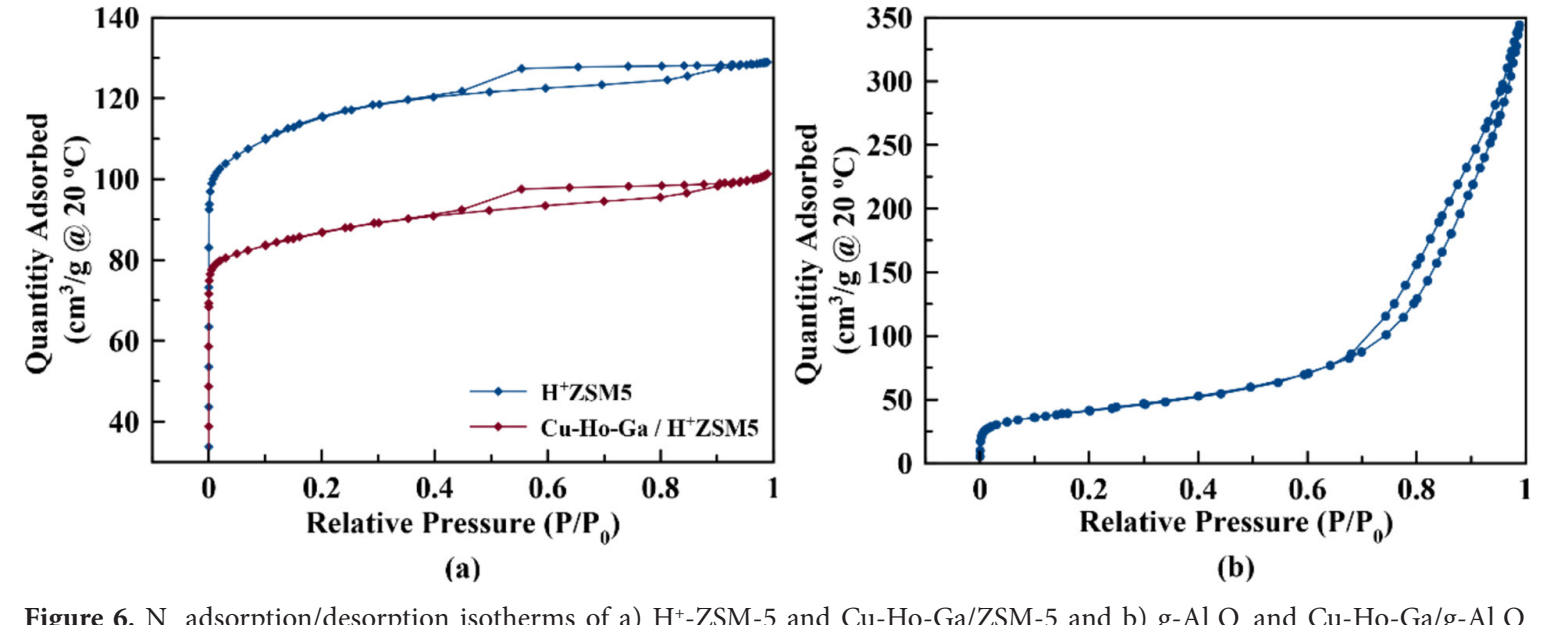
N_2_ adsorption/desorption isotherms of a) H+-ZSM-5 and Cu-Ho-Ga/ZSM-5 and b) g-Al_2_O_3_ and Cu-Ho-Ga/g-Al_2_O_3_ obtained at –196 °C.

N2 adsorption isotherms obtained at –196 °C on H+-ZSM-5, Cu-Ho-Ga/ZSM-5, /g-Al_2_O_3_, and Cu-Ho-Ga/g-Al_2_O_3_ can be seen in Figures 6a and 6b. The surface areas, total pore volumes, and micropore and mesopore volumes of the samples before and after the metal loading of H+-ZSM-5 and after the metal loading of g-Al_2_O_3_ are given in Table 4. H+-ZSM-5 contains both micropore volume and mesopore volume. Cu-Ho-Ga/g-Al_2_O_3_ contains mesopore volume. t-plot micropore volumes of pure H+-ZSM-5 and Cu-Ho-Ga/ZSM-5 were measured as 0.129 cm3/g and 0.104 cm3/g, respectively. Following the loading of Cu-Ho-Ga, it was observed that the volume of micropores as well as surface areas on H+-ZSM-5 decrease. This could be because of some of the dissolved Cu(II), Ho(III), or Ga(III) cations exchanged inside the micropore voids of the zeolites. 

**Table 4:** Surface area and pore volume values of H+-ZSM-5, Cu-Ho-Ga/ZSM-5, and Cu-Ho-Ga/g-Al_2_O_3_.

Sample	SBET (m2 / g)	Vtotal (cm3/g)	Vmicro (cm3/g)	Vmesoa (cm3/g)
H+-ZSM-5	376	0.199	0.129	0.070
g-Al_2_O_3_	176	0.656	0.009	0.647
Cu-Ho-Ga/ZSM-5	282	0.156	0.104	0.052
Cu-Ho-Ga/g-Al_2_O_3_	145	0.507	0.011	0.496

a: Vmeso = Vtotal-Vmicro

#### 3.2.3. Morphology and elemental analysis

**Figure 7:**
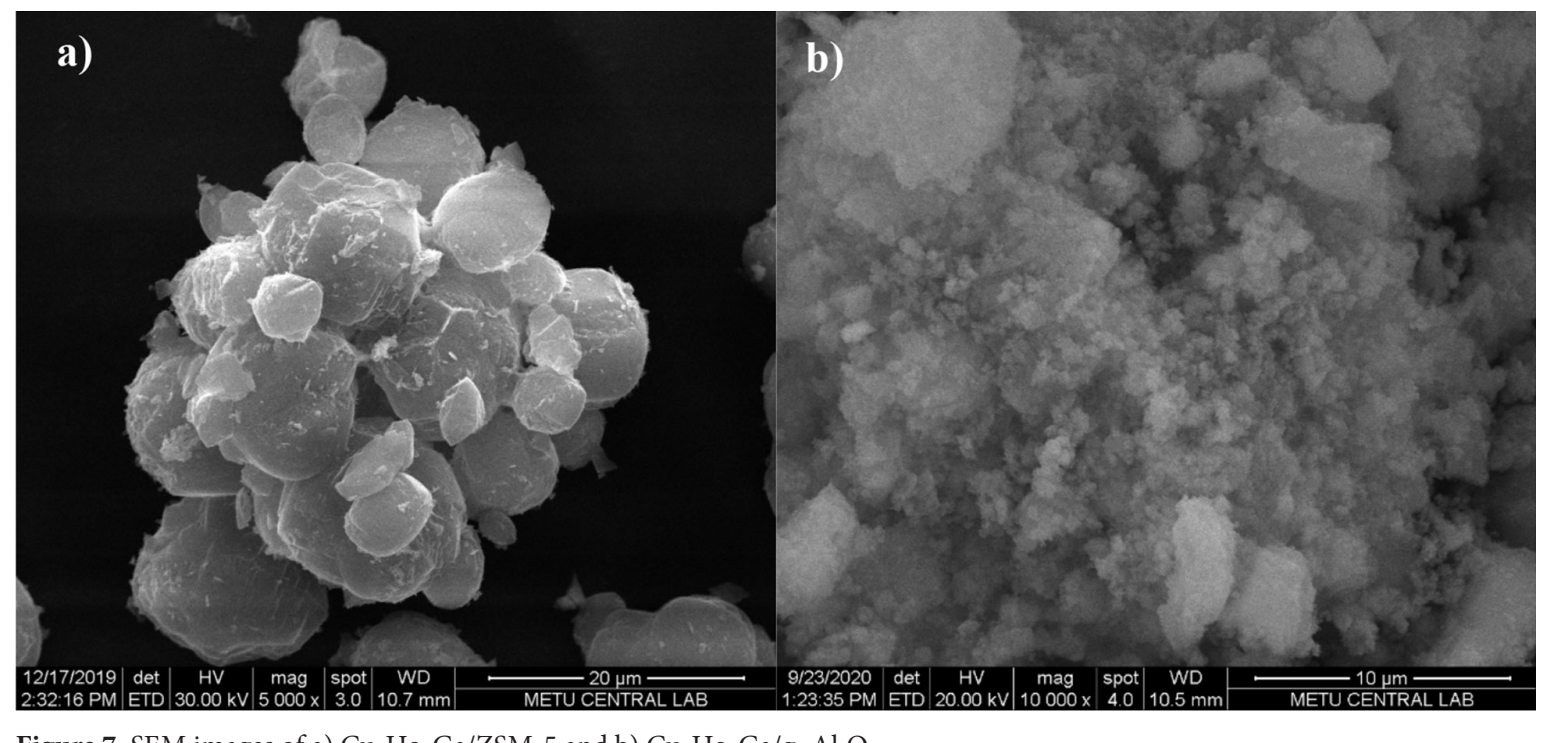
SEM images of a) Cu-Ho-Ga/ZSM-5 and b) Cu-Ho-Ga/g–Al_2_O_3_.

The scanning electron microscopy (SEM) image of Cu-Ho-Ga/ZSM-5 can be seen in Figure 7. ZSM-5 crystals have crystal sizes between 5 and 15 mm (Figure 7a) and Al_2_O_3_ having particle sizes approximately 200 nm (Figure 7b) without any clear evidence of metal clusters. When the elemental analysis was performed using energy dispersive X-ray spectrometry (EDX), the average weight percentages were found to be 9.7 wt.% for Cu, 4.8 wt.% for Ho, and 3.6 wt.% for Ga on ZSM-5, in agreement with the intended metal loading percentages: 10 wt.% Cu, 5 wt.% Ho, and 5 wt.% Ga. For Al_2_O_3_, weight percentages were found to be 15.9 wt.% for Cu, 10.6 wt.% for Ho and 4.7 wt.% for Ga.

### 3.3. Catalytic CO_2_ hydrogenation reaction results

The kinetic results of the reaction were obtained by performing reactions at 210 °C and 260 °C and at atmospheric pressure using ZSM-5- and g–Al_2_O_3_-based 0.35 g catalysts. According to these results, methanol, DME, and CO were observed as the main products with a small amount of CH4 formed.

By examining two different catalysts; Cu-Ho-Ga/ZSM-5 and Cu-Ho-Ga **/** g–Al_2_O_3_, at the same feed ratio (1:3 CO_2_:H_2_) and at the same temperature (260 °C), the total methanol and DME formation rates of g–Al_2_O_3_-based catalyst are nearly two times of that of ZSM-5-based catalyst (See Table 5). The reason for the lower methanol formation activity (per gram of the catalyst) on Cu-Ho-Ga/ZSM-5 could be due to the inactive Cu(II) or Ho(III), Ga(III) cations that are residing inside the micropores, which cannot be reduced as freely as the nanoclusters of CuO at the surface of the ZSM-5 crystals. 

**Table 5:** Methanol, DME, and CO formation rates on 0.5 g of Cu-Ho-Ga/H+-ZSM-5 and Cu-Ho-Ga/g-Al_2_O_3_at 260 °C and at 1 atm for a CO_2_:H_2_of 1:3.

Catalyst	CH3OH formation rate(µmolCH3OH/g_cat_/h)	DME formation rate(µmolDME/g_cat_/h)	CO formation rate(µmolCO/g_cat_/h)	Selectivity of CH3OH+DME (%)
Cu-Ho-Ga/g–Al_2_O_3_	41.7 ± 0.9	3.2 ± 0.2	6825 ± 400	0.7
Cu-Ho-Ga/ZSM-5	23 ± 1	5.6 ± 0.4	553 ± 56	5.9

**Figure 8:**
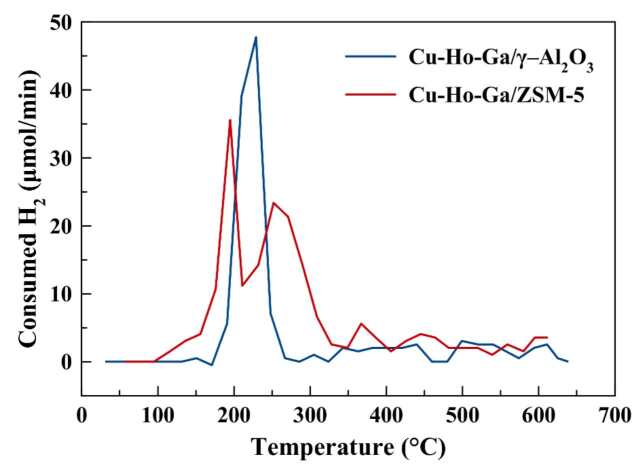
H_2_TPR analyses of Cu-Ho-Ga/ZSM-5 and Cu-Ho-Ga/g–Al_2_O_3_.

TPR experiments conducted on calcined samples show complete reduction of CuO on g–Al_2_O_3_ at 230 °C (see Figure 8), similar to TPR profile of commercial Cu/ZnO/g–Al_2_O_3_ catalyst [26]. On the other hand, for complete reduction of Cu(II) on ZSM-5, temperatures above 300 °C are needed on ZSM-5 (see Figure 8) [27]. Reduction of Ga2O3 and Ho2O3 are expected at temperatures higher than 500 °C [28,29]. Reduction at 250 °C reduces only 51% of the reducible metals on ZSM-5 (1051 μmol H_2_/g_cat_ out of total 2073 μmol H_2_/g_cat_), whereas this ratio is found to be 72% on g–Al_2_O_3_ (948 μmol H_2_/g_cat_ out of total 1311 μmol H_2_/g_cat_) based on TPR analysis.

Higher ratio of formation rates of DME over methanol at 260 °C were observed for ZSM-5-based catalyst when compared to g–Al_2_O_3_-based catalyst (see Table 5). The reason for this is stronger Bronsted acid sites on ZSM-5 as expected. The weaker acidity on g–Al_2_O_3_ causes lower methanol dehydration rates as well as lower water stability compared to ZSM-5 [30]. 

**Figure 9:**
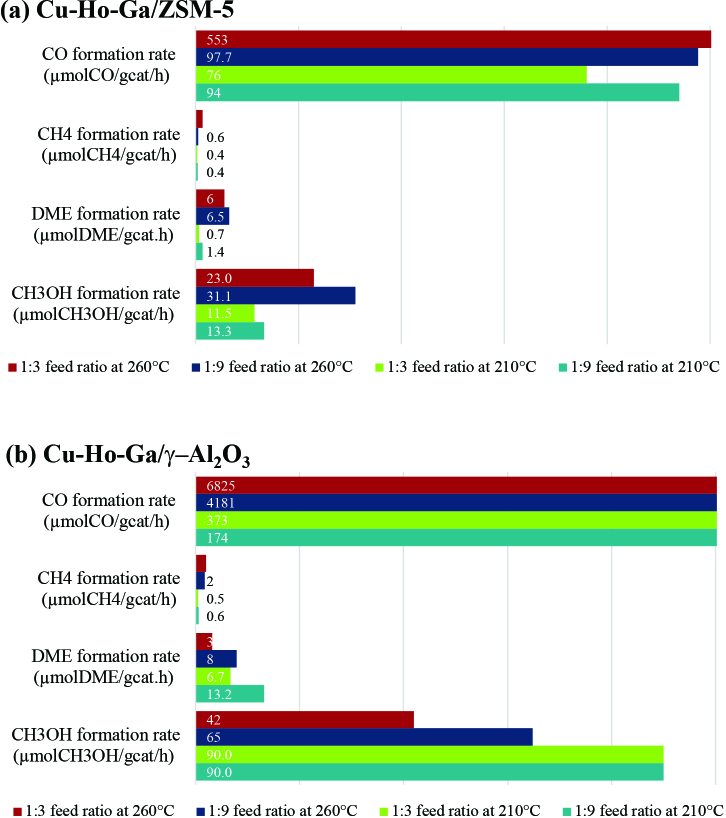
Product formation rates on a) Cu-Ho-Ga/ZSM-5 and b) Cu-Ho-Ga/g–Al_2_O_3_ at 210 and 260 °C and CO_2_:H_2_ ratios of 1:3 and 1:9.

Lower temperatures (210 °C) affected the methanol and DME formation rates differently in two different catalysts (see Figure 9). For Cu-Ho-Ga/ZSM-5, a decreasing trend in methanol and DME formation rates have been observed, which is opposite to what is observed on Cu-Ho-Ga **/** g–Al_2_O_3_. Decreased methanol and DME formation rates at 210 °C could be due to the slower CO_2_ hydrogenation kinetics. Alternatively, presence of water, which is produced all from CO_2_ hydrogenation, reverse water gas shift reaction, and methanol dehydration reactions, could cause blockage of the Bronsted acid sites as well as oxidation of Cu(0) metals into Cu(II) on zeolites [31,32] that would decrease the DME and methanol production. Especially at 200 °C, presence of water could cause competitive adsorption, similar to the case of NO oxidation, where decreased catalytic activity was reported due to the competitive adsorption of water and NO on Cu-ZSM-5 [33].

**Figure 10:**
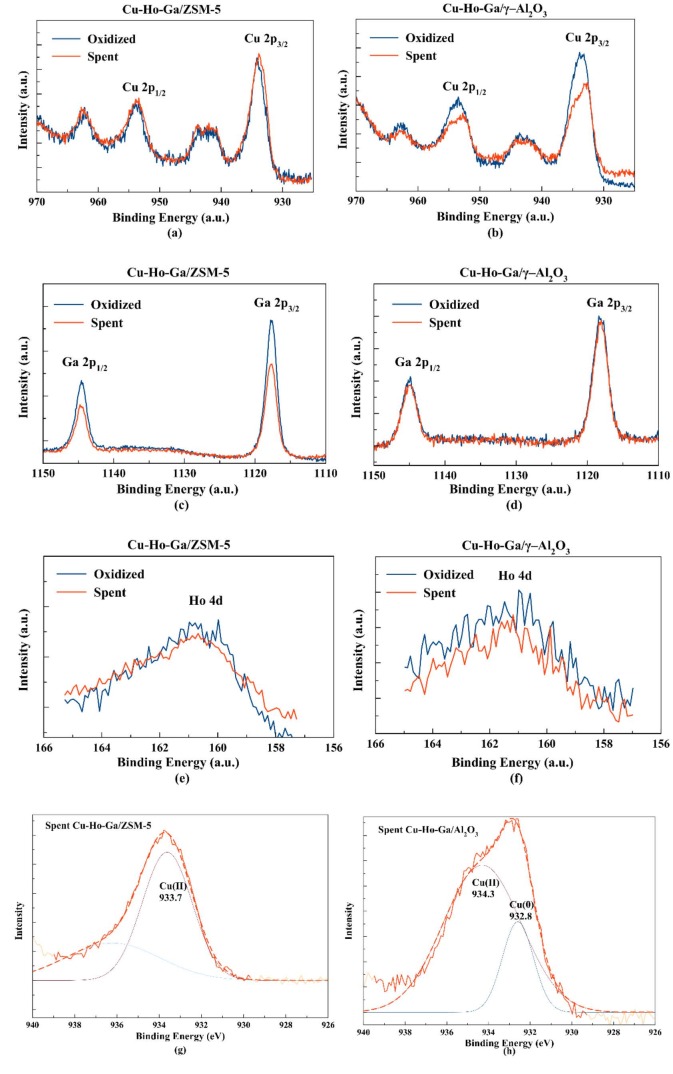
XPS analysis on oxidized and spent a,c,e) Cu-Ho-Ga/ZSM-5 and b,d,f) Cu-Ho-Ga/g–Al_2_O_3_. XPS Cu 2p3/2 region for spent g) Cu-Ho-Ga/ZSM-5 and h) Cu-Ho-Ga/g–Al_2_O_3_

XPS analyses of calcined and spent samples are given in Figures 10a–10f. A visibly higher Cu(II) fraction is evident on Cu-Ho-Ga/ZSM-5 when compared to that on Cu-Ho-Ga **/** g–Al_2_O_3_ (peaks at 933.7 eV and 934.3 eV for Cu(II) 2p3/2 and 932.8 eV for Cu(0) 2p3/2 [34], see Figures 10g and 10h), indicating either inefficient reducibility of Cu(II) as mentioned before, or oxidation due to the abundant production of water. Oxidized Ga and Ho were also observed (Ga 2p3/2 at 1118 eV for Ga2O3 [35] Ho 4d5/2 at 161.2 eV for Ho2O3 [36]) on spent catalysts that are exposed to air (see Figures 10c–10f).

In contrast to Cu-Ho-Ga/ZSM-5, increased methanol and DME formation rates are observed on Cu-Ho-Ga **/** g–Al_2_O_3_ at 210 °C with suppressed CO formation. This could be due to the exothermicity of the methanol and DME formation reactions as well as endothermicity of the reverse water gas shift reaction, favoring methanol and DME formation at lower temperatures. Increased methanol and DME formation rates sum up to 103 µmol/g_cat_/ h (90.3 µmolCH3OH/g_cat_/h and 13.2 µmolDME/g_cat_/h) at 210 °C and a CO_2_:H_2_ feed ratio of 1:9. Even though these values are lower than 5625 μmol_MeOH_/g_cat_/h obtained on Ni_5_Ga_3_/SiO_2_ at 210 **°** C and 1 atm (CO_2_:H_2_ = 1:3) [10], it is higher than 72 μmol_MeOH_ /g_cat_ /h methanol formation rate obtained on Cu/ZnO catalyst at 210 **°** C and 1 atm (CO_2_:H_2_ feed ratio of 1:9) [11].

For Cu-Ho-Ga/ZSM-5, major decrease in CO formation rate is observed by increasing the H_2_ content of the feed to a CO_2_:H_2_ feed ratio of 1:9 at 260 °C, in accordance with the thermodynamic expectance. At these conditions, a methanol formation rate of 31 µmolCH3OH/g_cat_/h and a combined methanol and DME selectivity of 31% are observed on Cu-Ho-Ga/ZSM-5, which is higher than reported methanol and DME selectivity of 15% on Ni_5_Ga_3_/SiO_2_ at 230 **°** C [9]. However, at 210 **°** C, the CO formation rate did not differ much from the value of 98 µmolCO/g_cat_/h obtained at 260 °C and CO_2_:H_2_ feed ratio of 1:9 (see Figure 9). Considering low CO formation together with decreased methanol and dimethyl ether rates, we can conclude that blockage of Cu and Bronsted acid sites rendered 210 °C an unfavorable temperature for all three reactions (Eqs. (1)–(3)) on Cu-Ho-Ga/ZSM-5.

On the other hand, effect of temperature and CO_2_:H_2_ ratio in the feed is clearly observable for Cu-Ho-Ga/g–Al_2_O_3_. At 210 °C, lower CO selectivity values can be achieved with higher methanol and DME formation rates. For a CO_2_:H_2_ ratio of 1:9, 59.8% CO selectivity and 30.9% methanol selectivity were observed using Cu-Ho-Ga/g–Al_2_O_3_ (see Table 6). At 260 °C a lower methanol selectivity value of 1.5% on Cu-Ho-Ga/g–Al_2_O_3_ was noted for the same CO_2_:H_2_ ratio, in accordance with the thermodynamic analysis. Therefore, we can conclude that lower temperature (210 °C) and 1:9 CO_2_:H_2_ resulted in higher methanol+ DME selectivity on Cu-Ho-Ga/g–Al_2_O_3_ (see Table 6). This is due to the exothermicity of reactions 1 and 3 and stoichiometric coefficients of the 3 main reactions in Table 1. The methanol and DME formation rates are expected to increase when the H_2_ ratio in the feed is higher. 

**Table 6:** The methanol + DME and CO selectivity values of 0.5 g of Cu-Ho-Ga/ZSM-5 and Cu-Ho-Ga/g-Al_2_O_3_at 210 °C and 260 °C and at 1 atm with different feed ratios.

Catalyst	Reaction temperature (°C)	CO_2_:H_2_feed ratio	SCH3OH (%)	SCH3OH+ SDME (%)	SCO (%)
Cu-Ho-Ga/g–Al_2_O_3_	210	1:9	30.9	39.9	59.8
		1:3	18.8	21.6	78.3
	260	1:9	1.52	1.9	98.1
		1:3	0.61	0.7	99.3
Cu-Ho-Ga/ZSM-5	210	1:9	12	14.5	85.2
		1:3	12.8	14.3	85.2
	260	1:9	21.8	31.0	68.6
		1:3	4.0	5.9	93.9

**Figure 11:**
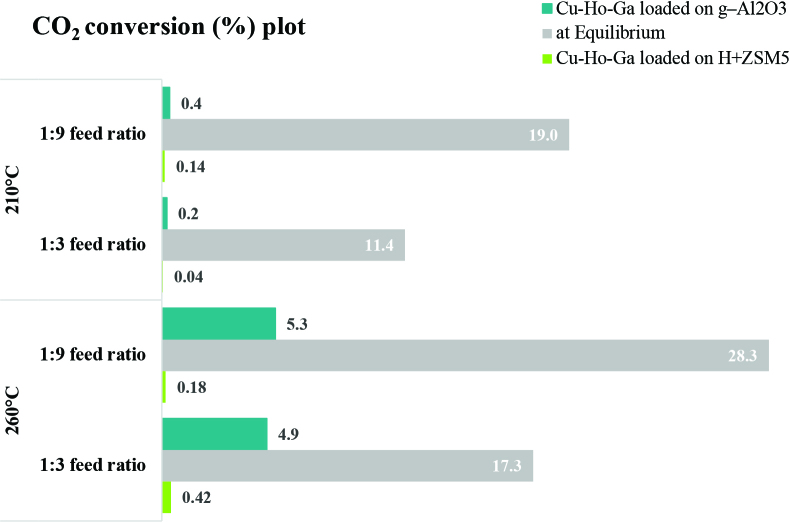
Experimental and equilibrium CO_2_ conversion (%) values on Cu-Ho-Ga/ZSM-5 and Cu-Ho-Ga/g–Al_2_O_3_.

CO_2_ conversion results are shown in Figure 11. According to the catalytic activity of both Cu-Ho-Ga/g-Al_2_O_3_ and Cu-Ho-Ga/ZSM-5, the highest conversion of CO_2_ (%) was recorded at 260 °C and at 1:9 CO_2_:H_2_ ratio. The calculated CO_2_ conversion values (such as 5.3% at 260 °C and at 1:9 CO_2_:H_2_ ratio on Cu-Ho-Ga/g-Al_2_O_3_) are lower than the calculated equilibrium conversion values (28% at 260 °C and at 1:9 CO_2_:H_2_ ratio), indicating room for improvement for the observed rates on these catalysts.

## 4. Conclusions

This study reports the kinetic and thermodynamic analysis of DME and methanol synthesis from CO_2_ hydrogenation reaction at atmospheric pressure. Cu-Ho-Ga/g-Al_2_O_3_ was found to be a more efficient catalyst with methanol formation rate reaching 90.3 µmolCH3OH/g_cat_/h and a DME formation rate of 13.2 µmolDME/g_cat_/h. A combined methanol and DME selectivity of 39.9% was observed at 210 °C and a 1:9 CO_2_:H_2_ feed ratio. Temperature increase affected the methanol formation rate and selectivity values on Cu-Ho-Ga/g-Al_2_O_3_ and Cu-Ho-Ga/ZSM-5 differently. On Cu-Ho-Ga/g-Al_2_O_3_, increased temperature resulted in decreased methanol formation and accelerated CO formation (and therefore much lower methanol selectivity) as expected from the thermodynamic analysis. However, for Cu-Ho-Ga/ZSM-5, 210 °C was found to be a low temperature for significant methanol and dimethyl ether production as well as reverse water gas shift reaction due to the inactivation of the Cu and Bronsted acid sites in presence of water vapor. Only at 260 °C, the reaction results followed thermodynamic trends by showing enhanced methanol selectivity with an increased H_2_ content in the feed. An increase in the methanol and DME selectivity from 5.9% to 31% was observed with decreased CO_2_:H_2_ ratio from 1:3 to 1:9. Higher CO_2_ conversions were observed at 260 °C with the highest value of 5.3% using a CO_2_:H_2_ ratio of 1:9. Optimization of the catalysts is encouraged for enhancing the observed methanol formation rates and selectivity values as the results were found to be away from the equilibrium. At the investigated conditions, the highest methanol formation rate and selectivity were found as 90.3 µmolCH3OH/g_cat_/h and 30.9%, respectively.
